# Displaced juvenile Tillaux fractures: Surgical treatment and outcome

**DOI:** 10.1007/s00508-016-1059-9

**Published:** 2016-08-17

**Authors:** Thomas M. Tiefenboeck, Harald Binder, Julian Joestl, Michael M. Tiefenboeck, Sandra Boesmueller, Christian Krestan, Mark Schurz

**Affiliations:** 1 Department of Trauma Surgery, Medical University Vienna, Waehringer Guertel 18–20, 1090 Vienna, Austria; 2 Department of Radiology, Medical University of Vienna, Vienna, Austria; 3 Department of Orthopaedics, Herz Jesu Hospital, Vienna, Austria

**Keywords:** Tillaux fracture, Operative treatment, Retrospective study, Open reduction, Foot and ankle score

## Abstract

**Background:**

Approximately 15 % of all juvenile injuries of the long bones involve the epiphyseal growth plate, and 2.9 % of these are juvenile Tillaux fractures. The Tillaux fracture is of great importance because it involves a major weight-bearing articular surface. Treatment protocols in the literature are not uniform for this kind of fracture, and numerous case reports can be found describing various treatment methods. The aim of this study was to present the clinical outcome at long-term follow-up after treatment of displaced Tillaux fractures.

**Methods:**

In all, 168 children and adolescent patients with physeal injuries of the distal tibia were treated from 2003 to 2012. Seven patients were identified as having Tillaux fractures requiring surgical treatment and therefore were included in our study and evaluated retrospectively.

**Results:**

Seven patients with Tillaux fractures underwent surgical reconstruction by open or closed reduction. Excellent results were achieved in 90 % of the patients, with a mean Foot and Ankle Score at the last follow-up of 98.71.

**Conclusion:**

Anatomical reduction is required for every displaced epiphyseal fracture via open reduction and internal fixation, especially in cases with ≥2 mm fragment displacement. Plaster cast immobilization and non-weight-bearing mobilization for at least 4 weeks might be a good way of ensuring optimal surgical results and preventing complications.

## Introduction

An isolated fracture of the anterolateral distal tibial epiphysis is called juvenile Tillaux fracture. Paul Jules Tillaux first described the avulsion fracture of the distal tibial physis in 1892 [1]. Approximately 15 % of all juvenile injuries of the long bones involve the epiphyseal growth plate [2], fractures of the distal tibial epiphysis account for 11–20 % to these injuries [3], and 2.9 % of these are juvenile Tillaux fractures [4]. Triplane and Tillaux fractures occur after supination external rotation and compression stress with unpredictable multiplanar fracture patterns [5]. The open region represents an area of weakness in the distal tibia with a risk for Tillaux fractures [6]. Because the fracture can appear different on X‑ray images, computed tomography is often necessary to determine the number of fragments [5, 7]. All of these fractures are intra-articular and therefore anatomic or near-anatomic reduction of the joint surface is recommended to minimize future posttraumatic ankle arthritis [5], joint stiffness, and pain [6]. The fracture is of great importance because it involves a major weight-bearing articular surface [8]. Most of these fractures occur at the end of growth, and growth arrest occurs rarely [5]. 

In the literature the treatment protocols for this kind of fracture are not uniform, therefore a great number of case reports can be found describing various treatment methods [8–10]. The treatment procedures of this fracture are based on the displacement. Nondisplaced or minimally displaced (<2 mm) fractures can be treated by immobilization with casts, whereas most of the authors conclude that for a displacement of over 2 mm, closed or open reduction is necessary to restore articular congruity [3, 11–13].

This study presents, to our knowledge, one of the largest single-center reports on displaced juvenile Tillaux fractures in recent literature focusing on the clinical–functional and radiographic outcome.

## Methods

From 2003 to 2012, 168 patients with physeal injuries of the distal tibia were treated at the Department of Trauma Surgery and evaluated for retrospective follow-up. Included were all patients who underwent primary treatment of physeal injuries at our Department of Trauma Surgery. Diagnosis was based on clinical and recognized radiological criteria. All patient information, disease-, and treatment-related data were retrieved by a review of the patients’ charts. Prior to the investigation, the corresponding institutional review board approved this study (EK Nr. 1216/2014).

**Fig. 1:**
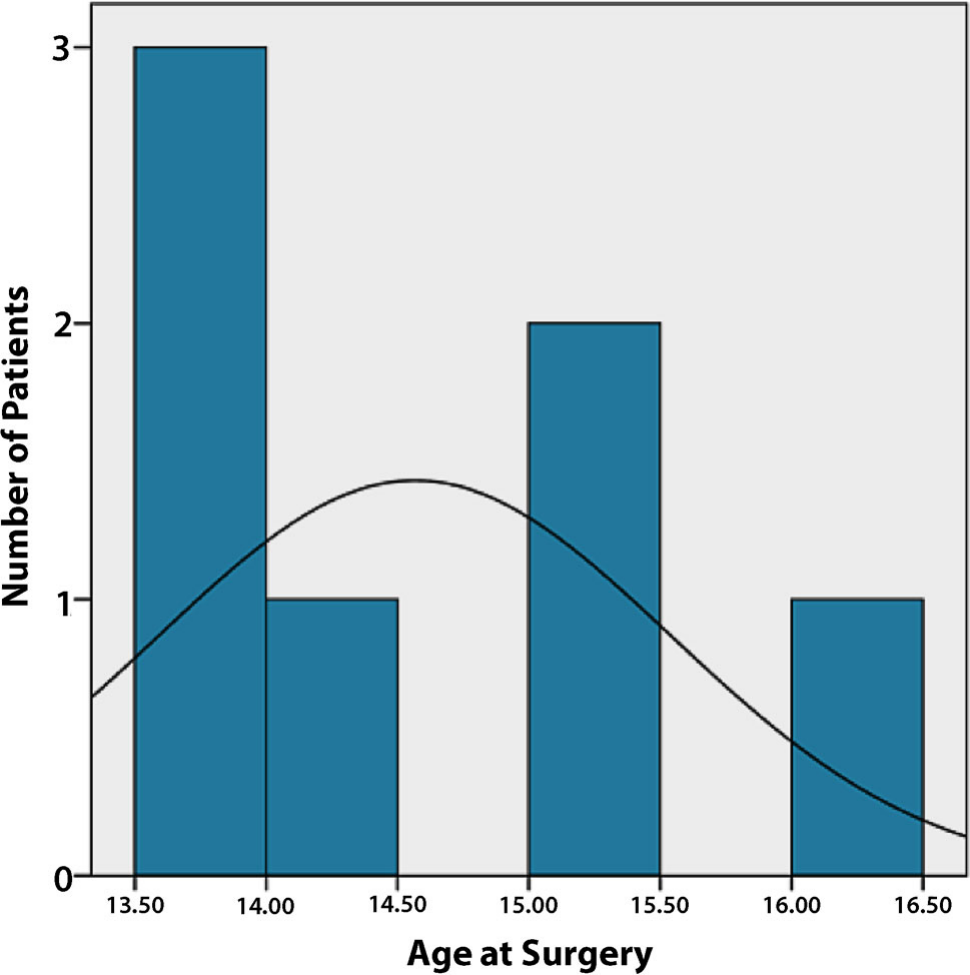
Overview of patients’ age at the time of surgery

Seven out of 168 patients met the inclusion criteria, comprising four male (57 %) and three female (43 %) patients with a mean age of 15 years (range, 14–16 years); details of the age distribution are presented in Fig. 1.

The first assessment involved a clinical examination, an X-ray in two planes, and in all but one patient a computed tomography (CT) study. An examination clarifying the state of the skin and the neurovascular status is necessary to indicate emergency surgery.

All patients were closely followed up until removal of the implants was possible. After screw removal, wound healing, and full recovery of function, there is no need to follow up patients closely and they were told to come back if complications occurred.

However, for this study all patients were invited for follow-up investigation by letter or telephone call. All patients signed informed consent forms before the investigation.

Data of five out of seven patients were available for a mean long-term follow-up of 79 months (range, 40.43–126.80 months).

Every clinical chart, X‑ray, and/or CT scan was reviewed. All X‑rays and CT scans were analyzed by two blinded investigators (one radiologist with 20 years of experience and one trauma surgeon with 14 years of experience) establishing the fracture type. In all patients, the Foot and Ankle Score [14] was recorded after a mean time of 6 months and at the last follow-up, after a mean of 79 months.

In this case series all but one fracture (>2 mm) was treated by open reduction and internal fixation (ORIF) to achieve anatomic reduction with additional plaster cast immobilization for at least 4 weeks (non-weight-bearing).

### Statistical analysis

Descriptive data (mean, median, range, proportions) are reported for the entire patient cohort. Statistical analysis focused on surgical, radiographic, and functional outcome after treatment of a Tillaux fracture. Therapeutic variables (surgery, adjuvant therapy, and function), pathological variables (refracture, complications), and demographic variables (sex, age and follow-up) were examined. All calculations were made using Microsoft Excel®, SPSS® software (Version 21.0, SPSS Inc., Chicago, Ill.).

## Results

All patients presented first with pain and swelling at the outpatient clinic, one patient presented additionally with a malposition of the ankle. After initial X‑ray and CT scan (86 %), a fracture dislocation (>2 mm) requiring operative fracture reduction was found in all patients. Therefore, screw fixation was used for all patients. Six fractures (86 %) needed ORIF; one (14 %) could be treated by percutaneous fracture reduction and internal fixation (CRIF). All patients were treated with an over-knee plaster cast immobilization after the surgical procedure for at least 4 weeks (range, 4–8 weeks). In all but one patient non-weight-bearing mobilization was carried out for 4 weeks; for the rest with cast immobilization partial weight-bearing was allowed in all patients (see Table 1 for detailed patient information).

**Table 1:** Overview of patient characteristics

Case no.	Sex/Age (years)	Cause	Foot and Ankle Score <1 year	Foot and Ankle Score >3 years	Treatment method	Length of follow-up in months	Cast immobilization in weeks	CT	Complication
1	M/16.27	Sport injury	100	100	ORIF	52.8	8	Yes	–
2	F/13.82	Sport injury	91	100	ORIF	126.8	5	Yes	–
3	F/13.67	Sport injury	100	100	ORIF	40.4	5	Yes	–
4	M/15.26	Sport injury	91	n.e.	CRIF	4.3	6	Yes	Limitation of ankle movement
5	M/14.15	Sport injury	95	100	ORIF	73.7	6	No	–
6	M/15.00	Accident	100	n.e.	ORIF	7.0	4	Yes	–
7	F/13.79	Sport injury	100	100	ORIF	101.6	4	Yes	–

*M *male*, F *female*, n.e.* not evaluated, *CT* computed tomography, *ORIF* open reduction internal fixation, *CRIF* closed reduction internal fixation

Overall six patients (86 %) had full and immediate recovery without suffering any complications, with a mean Foot and Ankle Score after 4 months of 96.71 points (range, 91.00–100.00; median 100.00). The Foot and Ankle Score improved over time and could be evaluated after a mean of 79 months in five patients, presenting with 100 points at the latest follow-up.

One patient presented with pain and joint stiffness during the follow-up period. The limitation of the ankle joint of 15° was treated conservatively by physical therapy and disappeared over a short time period. At the end of the study, the patient was free of complications and satisfied with the operative result.

Analyzing the trauma mechanism, we found six injuries occurring during sporting activities and one injury uring a walking accident.

At every follow-up date, X‑rays in two planes were made to assess fracture healing and to check for osteosynthesis. Additional clinical examinations were performed. A trauma surgeon specialized in children performed follow-up investigations. Patients were followed up very closely every week until cast immobilization was over and then at 3‑month intervals until full mobilization was possible.

In five patients (71 %) the screws were removed after a mean time of 8 months (range, 6.07–8.93 months; median 8.13 months). Osteosynthesis material was removed because of the patients’ and parents’ wish.

One patient moved back to the USA and another patient moved back to Russia before screw removal; both were satisfied with their operative results and presented with excellent clinical/functional outcome at the latest follow-up.

**Fig. 2:**
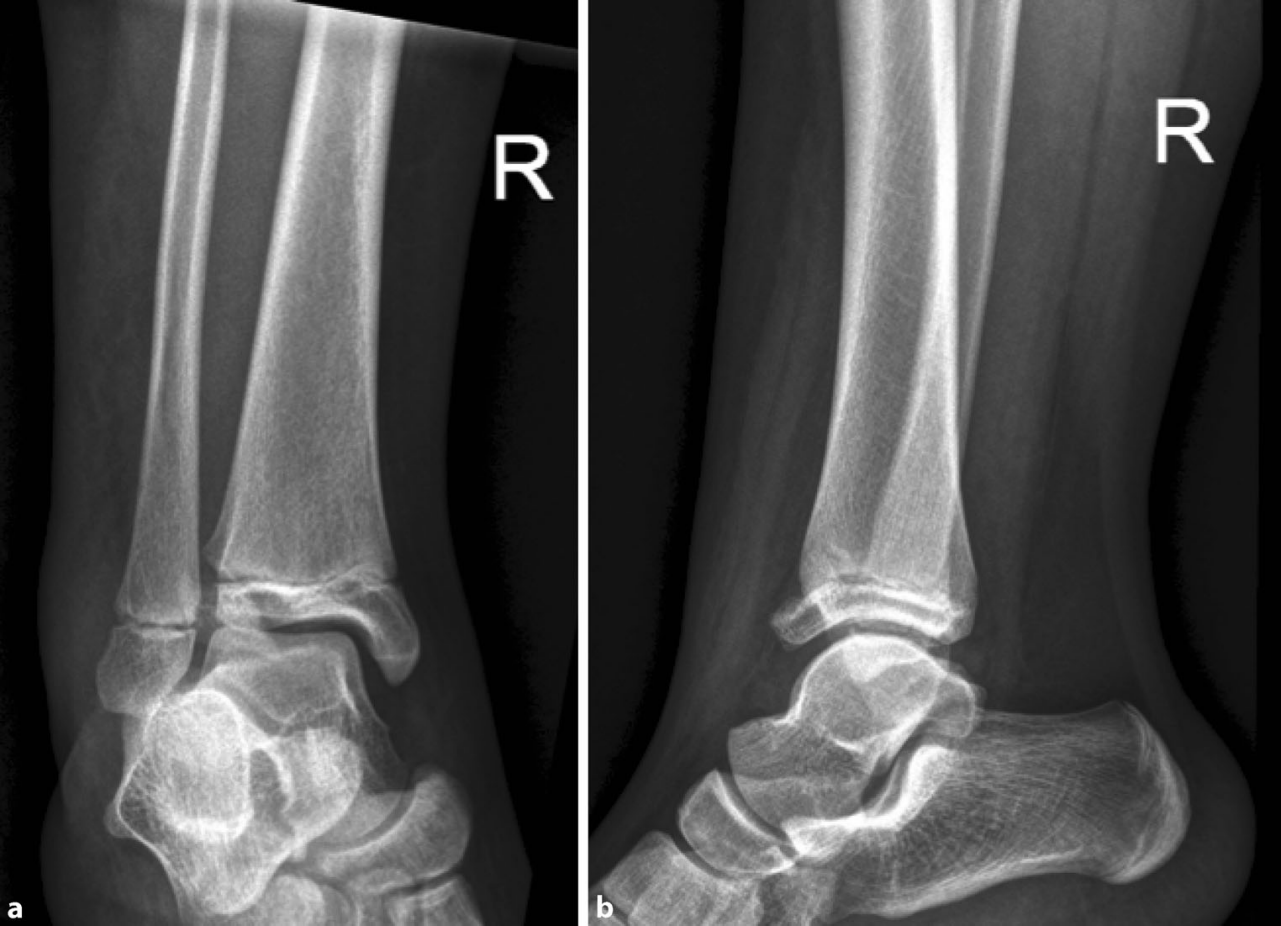
Initial X‑ray in two planes of a Tillaux fracture in a 14-year-old patient (**a** anteroposterior view, **b** lateral view)

**Fig. 3:**
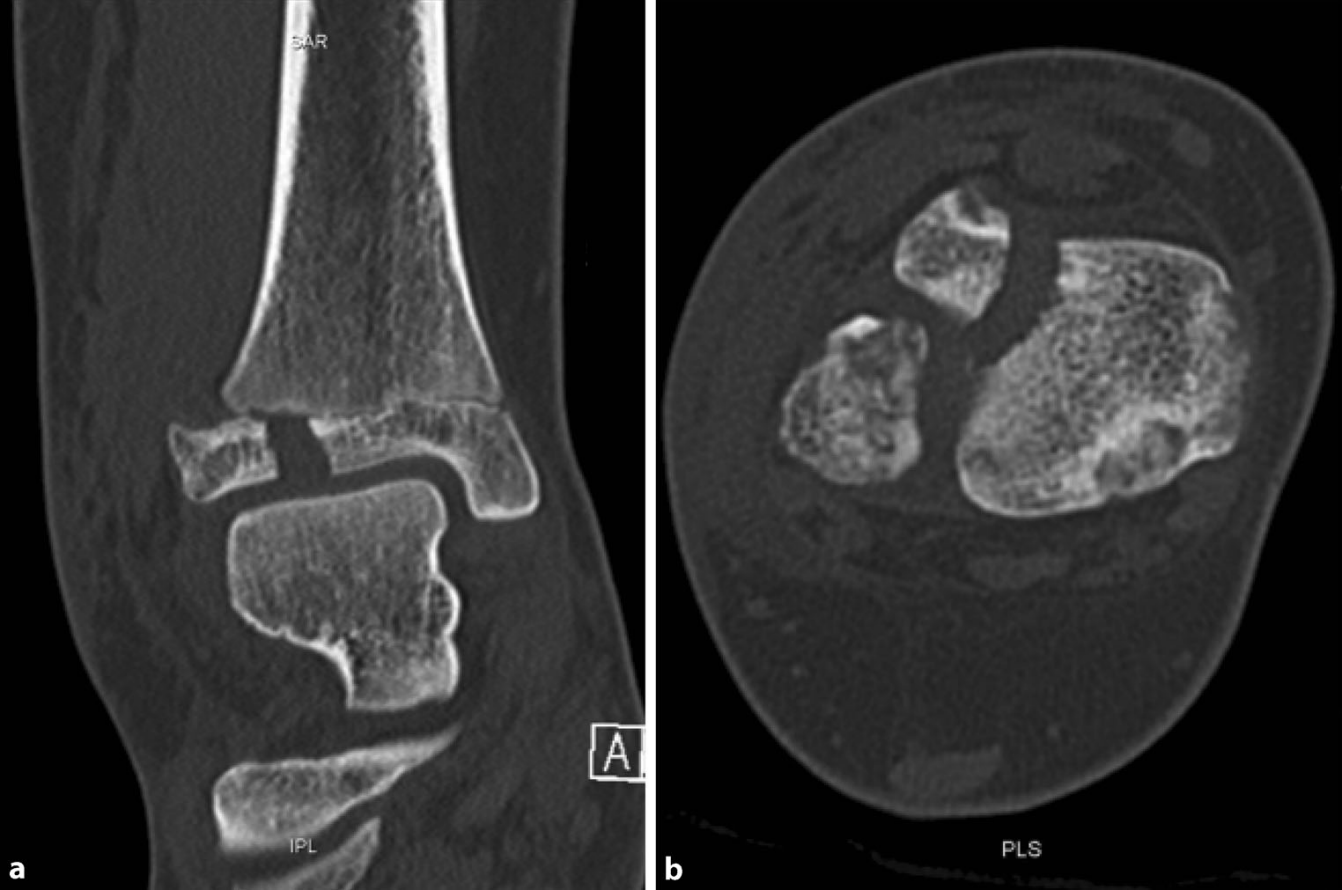
CT scan, coronal (**a**) and axial plane (**b**) of a Tillaux fracture in a 14-year-old patient

**Fig. 4:**
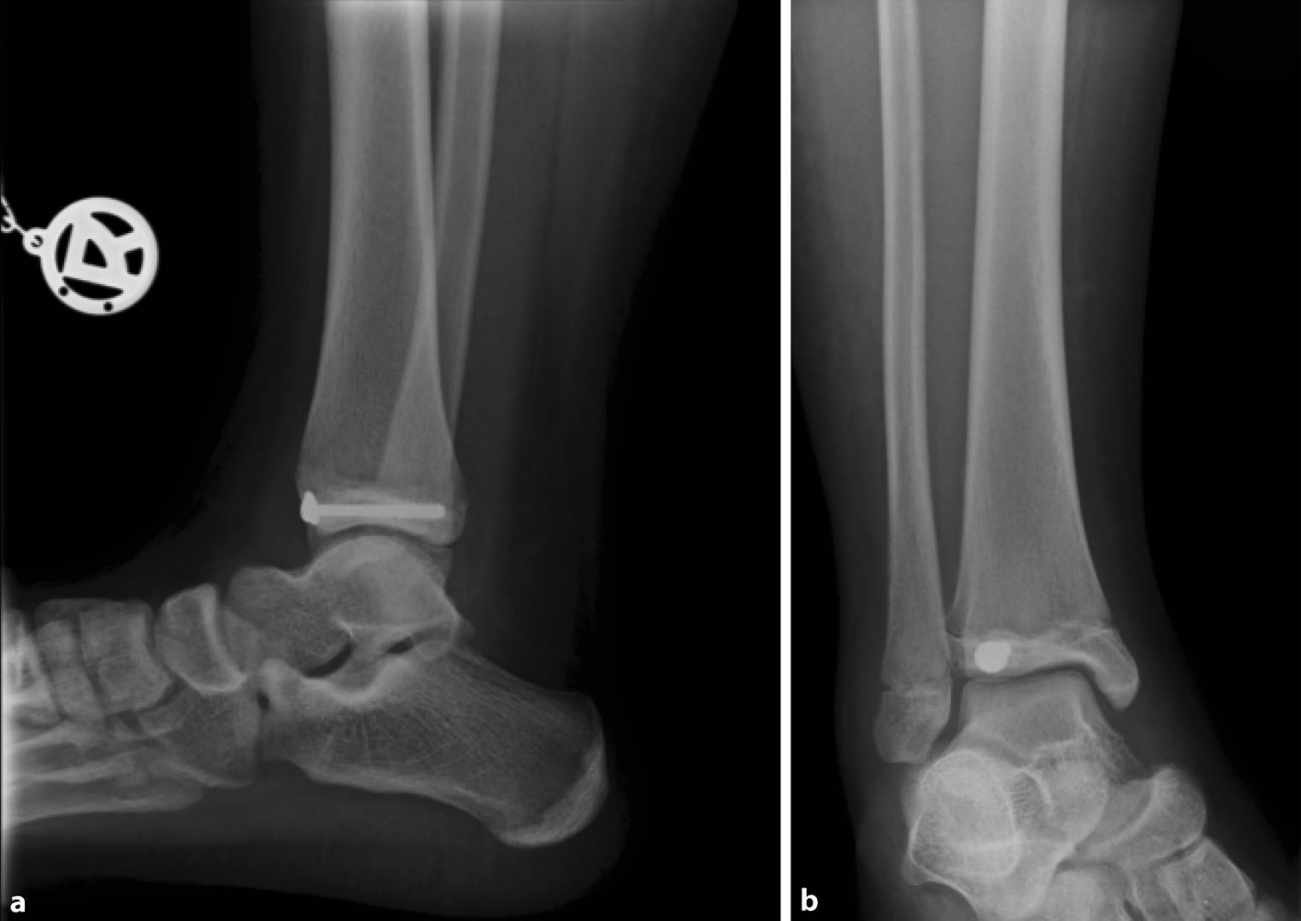
X-ray in two planes after open reduction and screw fixation in a 14-year-old patient (**a** anteroposterior view, **b** lateral view)

**Fig. 5:**
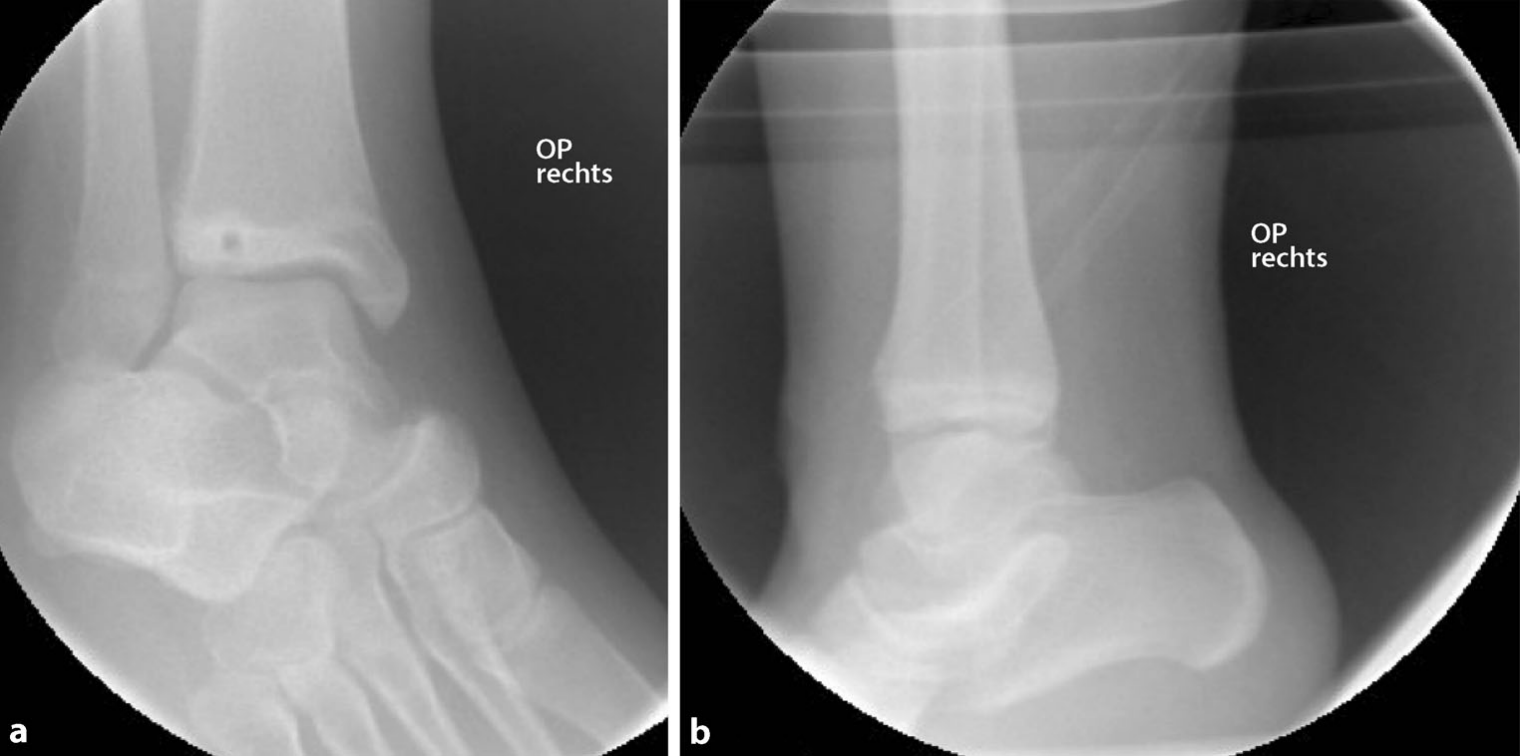
X-ray in two planes after screw removal in a 14-year-old patient (**a** anteroposterior view, **b** lateral view)

An exemplary case of a 14-year-old patient with a surgically treated Tillaux fracture is presented in Figs. 2 to 5 (Fig. 2a, b, X‑ray; Fig. 3a, b, CT scan; Fig. 4a, b, X‑ray after open reduction and screw fixation; Fig. 5a, b, X‑ray in two planes after screw removal).

## Discussion

The juvenile Tillaux fracture is defined as a fracture of the anterior distal tibial tubercle only occurring in adolescents. Paul Tillaux first described this fracture type from cadaveric studies in 1892 [1]. Today this fracture type is classified as a Salter–Harris type III fracture of the distal tibial epiphysis and is only seen in adolescents. Tillaux fractures and triplane fractures are the most common ankle joint fractures in adolescents with closure of the epiphyseal plate according to the mechanism of injury [5].

A review of the literature revealed only a few studies [5, 11–17] dealing with this type of fracture and most of them were case reports or case series.

We report on one of the largest case series of patients with displaced Tillaux fractures treated in one single institution. In summary, our study shows that ORIF and postoperative plaster cast immobilization lead to excellent results in displaced Tillaux fractures. There are certainly considerable limitations to this study, mainly its solely retrospective design, small cohort, and long duration of treatment, all explained by the nature of this injury. However, we used homogeneous treatment guidelines for all patients, but it is not possible to make a categorical statement because of the solely retrospective nature of the study and the small number of patients.

Owing to the low patient number, only a descriptive statistical analysis was performed. All patients presented at the last follow-up examination with excellent clinical results according to movement and weight-bearing, and therefore no further follow-up investigations were planned. Based on this knowledge, the data analysis has to be reviewed critically since it might overlook the risk of long-term complications.

All of our patients presented with an excellent foot and ankle score at the last follow-up (mean Foot and Ankle Score, 98.71). Diagnosis was made using X‑rays in two planes and additional CT scans to assess the extent of damage of a comminuted fracture and the articular surface, which is the standard procedure for diagnosing this injury [6, 18].

Some authors prefer ORIF of fractures with more than 2 mm of displacement [11, 19], which is in accordance with the general principles of treatment and was also done in our series. Except for one patient, all patients in our study were treated by ORIF and additional postoperative plaster cast immobilization. If possible, closed reduction and internal fixation can also be used for treatment [13], but was only performed on one of our patients.

Depending on patient’s age, owing to the long study period (10 years) and finally surgeons own decision the time of plaster cast immobilization varied between 4 and 8 weeks. All patients were treated with non-weight-bearing mobilization for at least 4 weeks with plaster cast immobilization to secure treatment outcome, although this is not described as standard treatment in the literature. We prefer an overall treatment with plaster cast immobilization to avoid compliance problems and secure good results as is also described by different authors in the literature [11, 20].

The complication rate of closed or open reduction with internal fixation is very low, as in our study. The lack of complications after surgery at the longest follow-up suggests that open reduction with internal fixation is a safe procedure for treatment of this type of fracture [21].

In all but one patient, ORIF was used, which is a safe procedure, as mentioned earlier, and justifies the short follow-up time if patients present with excellent clinical results after a mean of 6 months (mean Foot and Ankle Score 96.71 <1 year postoperatively). However, in five patients long-term follow-up data were available, showing excellent results with a Foot and Ankle Score of 100 and satisfaction in all patients.

In the literature, arthroscopic-assisted reduction and fixation of these fractures is also an established treatment option. By arthroscopy, direct visualization of the fragment and interpretation of associated chondral injuries is possible. Reviewing the literature we only found cases series describing this method and therefore we did not use it in our patients routinely [8, 22, 23].

To the best of our best, after reviewing the current literature (PubMed and Cochrane Library) we found only eight studies dealing with Tillaux fractures and numerous case reports. Table 2 presents the results of the studies in detail excluding case reports.

**Table 2:** Overview of the current literature

Author and year	Patients (*n*)	Treatment	CT	X-ray	Complications	Outcome	Cast immobilization	Follow-up	PDF available
Choudhry I. et al. 2013 [13]	20	ORIF and CRIF	–	–	–	Excellent, Foot and Ankle Score, Marx Activity Score	–	>2 years	No
Gourineni P. et al. 2011 [20]	8	ORIF	Yes (7/8)	Yes	In 3 patients; Achilles contracture; broken screws	No	4 weeks	>1 year	Yes
Dias L.S. et al. 1983 [24]	8	–	–	–	–	–	–	–	No
Cottalorda J. et al. 2008 [21]	30	ORIF	–	–	–	–	–	Mean 3 years	No
Kaya A. et al. 2007 [11]	10	ORIF	Not needed	Yes	10° loss of plantar flexion in 2 patients, in 1 patient limitation of recreational activities	AOFAS mean 99.3	6 weeks	Mean 54 months	Yes
Schlesinger I. et al. 1993 [10]	6	CRIF	Not mentioned	Yes	None	–	–	–	Yes
Stefanich R. et al. 2007 [3]	5	ORIF and conservative (1)	–	Yes	–	Excellent, No score used	Not mentioned	1 to 9 years	No
Kling T. et al. 1984 [25]	32 (combined Salter Harris III and IV)	Conservative therapy –(9) and ORIF	–	–	Growth disturbance (5/9)	Excellent No score used	–	–	No
Current Study	7	ORIF and CRIF (1)	Yes (6/7)	Yes	Wound healing disturbance; 15° loss of flexion	Excellent, Foot and Ankle Score	4 to 8 weeks	Mean 58 months	–

^a^ *CT* computed tomography, *ORIF* open reduction and internal fixation, *CRIF* closed reduction and internal fixation

The fracture location, degree of displacement, and the child’s age determine the correct treatment of pediatric ankle fractures. We claim to reduce epiphyseal injuries anatomically in every case by open means in accordance with the existing literature [11, 21].

Our results underline that every Tillaux displacement exceeding more than 2 mm should be corrected anatomically at every age to achieve excellent results.

## Conclusion

Anatomical reduction is required in every displaced Tillaux fracture, especially in cases with ≥2 mm fragment displacement.

Our results highlight that only an absolute anatomical fracture reduction and internal fixation can prevent a bone bridge and a consecutive epiphysiodesis with axial malalignment and limb length discrepancy, resulting in excellent clinical and radiological results. Plaster cast immobilization and non-weight-bearing mobilization for at least 4 weeks might be a good tool to ensure surgical results and prevent complications.
